# Permanent maxillary canine eruption in children with clefts – a retrospective study

**DOI:** 10.2340/aos.v85.45420

**Published:** 2026-02-11

**Authors:** Mateusz Podleśny, Riitta Lithovius, Leena Ylikontiola, George K. Sándor, Ville Vuollo, Virpi Harila

**Affiliations:** a Research Unit of Population Health, Faculty of Medicine, University of Oulu, Oulu, Finland; b Oulu University Hospital, Medical Research Center Oulu, Oulu, Finland

**Keywords:** Cleft lip, cleft palate, cleft lip and palate, canine eruption, alveolar bone graft

## Abstract

**Objective:**

The purpose of this study was to investigate the effect of alveolar bone grafting (ABG) on the eruption timing and the need for deliberation of the permanent maxillary canine in cleft lip and/or palate patients.

**Materials and methods:**

The study group included 45 cleft sites from a total of 39 unilateral cleft lip and alveolus (CLA, *n* = 5) and unilateral (*n* = 22) cleft lip and palate (CLP) and bilateral (*n* = 12) CLP patients who had undergone treatment in the Oulu Cleft Center (OCC). A split-mouth approach and linear mixed-effects regression analysis was used to compare findings between single individual grafted and non-grafted alveolar sites.

**Results:**

The maxillary canine angulation had a significant role in the timing of the canine eruption (estimate 0.06, 95% CI 0.04 to 0.08). While ABG seemed to delay the canine eruption, this result was not statistically significant. The eruption of the maxillary canine tooth on the bone grafted side was delayed compared to the eruption of the maxillary canine on the non-grafted side (estimate -0.55, 95% CI -0.99 to -0.11). Another factor affecting the canine eruption was surgical exposure of the canine, and it typically emerged about a year after it had been surgically exposed and deliberated (estimate 1.05, 95% CI 0.40 to 1.70).

**Conclusion:**

The permanent maxillary canine eruption was slightly delayed after the ABG. Also, the maxillary canine was found to typically emerge about a year after it has been deliberated. The early bone graft had no benefit in terms of the timing of canine eruption. The angulation of the maxillary canine should be taken into consideration when planning ABG to facilitate its eruption. Careful planning and timing of the ABG and multidisciplinary approach have an important role in cleft patients’ treatment.

## Introduction

Persistent intraosseus position of a tooth after the expected time of eruption can be defined as tooth impaction. Impaction can be evaluated clinically and radiographically using a dental panoramic tomogram and 3D imaging can be used to evaluate position and tooth resorption [1, 2]. Adjacent roots of the lateral incisors with optimal length tend to guide the maxillary canine to its correct place in mesially erupting canines. Having a missing lateral incisor results in a 2.4 times greater risk for palatal impaction of the maxillary canine [3].

The incidence of maxillary canine impaction varies between 0.8 and 5.2% depending on the population studied [4]. Maxillary canine impactions are twice as common in females as in males [5]. The incidence of maxillary canine impaction in unilateral cleft lip and palate (CLP) patients is approximately 10 times higher compared to the general population [6, 7]. The impaction rate following alveolar bone grafting (ABG) is reported to be between 12 and 35% [8]. Teeth tend to erupt until their root apices are closed. After apical closure, teeth lose their potential to erupt [9]. The causes for tooth impaction can be seen in Table 1.

**Table 1:** The most common causes for canine impactions according to Bishara and Ortho [5] and Sajnani et al. [10].

Tooth-related factors	Other local factors
Tooth size	Arch length discrepancies
Abnormal position of the tooth bud	Prolonged retention of the deciduous canine
Ankylosis	Early loss of the deciduous canine
Dilaceration of the root	The presence of an alveolar cleft
Iatrogenic origin	Cystic or neoplastic formation
	Idiopathic conditions

Alveolar clefts are usually formed between the lateral incisor and the maxillary canine [11]. Approximately, 37.6–58.6% of CLP patients are missing the lateral incisor congenitally on the cleft side [2]. This congenital absence of the maxillary lateral incisor is associated with up to 72.2% of cases of canine impaction in CLP patients [8]. Maxillary canine impaction is more frequent in CLP patients, especially on the cleft side. This may occur due to the higher vertical position of the maxillary canine as well as to delayed root development. Also, the erupting canine position is not guided by the lateral incisor when it is missing. The maxillary canine impaction can also be associated with a Class II malocclusion due to mesialisation of the teeth on the cleft side [1]. In the Finnish population, the normal eruption age for the maxillary canine ranges between 10 and 12 years of age [12].

Despite the Eurocleft Project 1996–2000, there does not seem to be an exactly standardised protocol for treating cleft patients. However, the Eurocleft Project attempts to provide a protocol that works well in Scandinavia [13]. ABG is performed after the eruption of the maxillary central incisor in the cleft region. ABG should be considered to stabilise the maxillary arch, allow canine eruption and sometimes facilitate the eruption of the lateral incisor [14, 15]. Some studies suggest bone grafting before the eruption of the central incisor with the aim of achieving better support for the dentition [16]. On average, ABG worldwide has been performed between 8.5 and 22 years of age, with the median being 14.45 years. The ABG has been performed routinely prior to the eruption of the maxillary canine in 90% of European and North American cleft teams. Better outcomes have been observed in ABG procedures if the maxillary canine root was approximately half developed [17]. In Finland, ABG is usually performed between 9 and 12 years of age before the eruption of the canine, but this has not been studied further on the Finnish population [18, 19]. However, the ABG timing based on patient age is inferior compared to the dental development stage [20]. According to a study by Jabbari et al., the outcome of alveolar bone graft in terms of bone height is not correlated to the original width of the cleft. However, the cleft width is associated with central incisor rotation and enamel hypoplasia [21].

There are many potential anatomic sites for harvesting autogenous bone grafts. Anterior iliac crest is widely used, as well as tibia, mandible, rib and cranial bone. Extraoral bone graft harvesting diminishes early and late morbidity. It is superior to other sites [14]. Grafts from the iliac crest have a high success rate with only a low incidence of complications [18]. In adults, autogenous bone can be harvested intraorally from the mandibular symphysis, mandibular ramus, maxillary tuberosity and zygomatic bone. The mandibular symphyseal alveolar graft provides sufficient amount of bone for an alveolar graft [22]. It is also associated with high patient satisfaction rate. The mandibular symphyseal alveolar graft is predictable and safe, and the area is easily accessible [23]. However, with large defects, it is better to harvest bone from the extraoral sites [24, 25].

Late bone grafting has a lower rate of success if the maxillary canine has already erupted [14]. Additional positive effects on secondary bone grafting are improvement of speech, facial appearance, fistula closure and oral hygiene [14]. According to Lique-Martin et al., there is no single specific protocol, which would ensure the success of the ABG procedure [18].

The maxillary canine angulation is associated with the possibility of impaction [26]. Cleft patients have an increased risk for maxillary canine impaction depending on the erupting canine position. The angulation of the maxillary canines is not affected by the bone grafting procedures [15]. However, the timing of ABG can prevent the impaction of the maxillary canine [27]. The original cleft width prior to grafting does not impact the success rate of the ABG. However, it may be used to indicate the future need for more extensive orthodontics [21].

The aim of this study was to determine whether ABG causes the delay of the eruption of the maxillary canine. Another aim of this study was to determine the need for surgical exposure of the maxillary canine after ABG. The working hypothesis was that the alveolar grafting procedure delayed the eruption of the maxillary canine.

## Materials and methods

**Figure 1:**
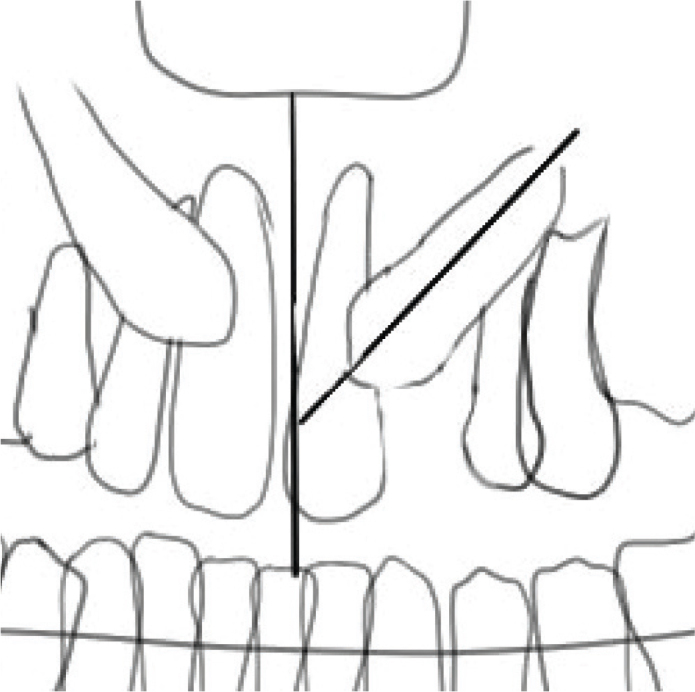
An illustration determining the canine angulation compared to vertical maxillary midline. The vertical plane is drawn through the nasal spine and midsagittal suture, and canine angulation is related to its axis compared to the vertical plane.

This study is a retrospective study based on patient’s hospital records. The study group consisted of 39 cleft patients with a total of 45 cleft sites. The patients were born between 2004 and 2013. All patients were treated in the OCC with an ABG during adolescence. Cleft lip and cleft alveolus patients who were evaluated for the need of ABG were included in this study. Patients without the need for ABG were excluded from the study population, as well as patients who had moved abroad or to different hospital district during their treatment time. All the unilateral cleft patients had their clefts occurring on the left side. The clefts were located between incisor and first premolar, mesial of the maxillary canine. The incisors were erupted. The lateral incisor was either missing or malformed. The same surgical team was responsible for all of the patients’ oro-facial surgical procedures since primary lip closure. The careful multidisciplinary treatment planning takes place at OCC by the same team. However, the orthodontic treatment is done in a variety of venues depending on where the patient lives. The need for ABG and surgical maxillary canine exposure was evaluated by the OCC. The criteria for ABG were the following: the maxillary canine root development greater than 50% as well as sufficient transversal and sagittal proportions. An earlier bone graft was performed in patients where the maxillary incisor was found to be malpositioned in an unfavourable inclination and therefore could not be orthodontically treated without bone graft. Eight bone grafts were performed before the age of nine, including two patients under the age of eight. The detailed distribution of clefts in this study can be seen in Table 2. An orthopantomogram was used to determine the angulation of the erupting maxillary canines. The vertical maxillary midline was used as a reference plane (Figure 1). There is no CT data of the majority of patients, so the bucco-palatal dimensions are not addressed in this study.

**Table 2:** Prevalence of different cleft types and sex in the study group.

Cleft type	*M*	*F*	Bilateral	Unilateral	Total
CLA	5	0	0	5	5
CLP	24	10	12	22	34
Total	29	10	12	27	39

M: male; F: female; CLA: cleft lip and alveolus; CLP: cleft lip and palate.

All of the patients were treated by an orthodontist to facilitate space for the erupting teeth, expand the maxilla and correct the lateral crossbite. The maxillary arches were expanded using orthodontic appliances in order to provide appropriate transversal relations. Every patient had orthodontic treatment before bone grafting. The eruption of 26 canines was documented.

### Statistical methods

R (R version 4.3.2) was used for statistical analysis. A split-mouth approach was used to compare findings in single individuals between grafted and non-grafted alveolar sites. The possible impact of the bone graft or the age at which it was performed on the eruption age was explored using regression analysis. To increase the sample size, data from both canines of individuals were included in the analyses. Due to potential patient-wise dependence, linear mixed models (LMM) were used, which account for these possible correlations in the random intercept. All models included sex, canine angle and canine deliberation as confounding variables. The first model included all teeth, while the second model focused on the teeth of unilateral patients. In both models, the exposure variable was the presence of a bone graft, and the outcome variable was the age of tooth eruption. The third model included all teeth from the grafted site. The exposure variable was the age at bone graft, and the outcome variable was the time between the bone graft and tooth eruption. This model design was also applied using mixed effects Cox regression. *P*-value of 0.05 was chosen as a threshold for statistical significance.

## Results

The average eruption age for the cleft side canine in this series was determined to be 11.68 years, and for the non-cleft side, it is 11.37. The mean age for the cleft side bone grafting was 9.69 years, with the age ranging between 7.83 and 12.25 years. Eight patients needed surgical canine exposure (20.5%). A total of nine cleft side canines and one non-cleft side canines were surgically exposed. Two of these patients had a bilateral cleft.

ABG has a tendency to delay the eruption of the respective maxillary canine when compared to the same patient’s contralateral maxillary canine and to other individuals. However, the results were not statistically significant. The eruption delay after ABG seemed to be between 3 and 6 months in this study group. In addition, the greater the maxillary canine angulation was, the slower the canine erupted. Having an angulation increase by 10 degrees delayed the respective canine eruption by 6 months (*p* ≤ 0.01 (Tables 3–5)). The later the bone graft was performed, the faster the canine erupted (*p* = 0.017 (Table 5)). A surgically exposed and deliberated canine erupted 1.05 years after the deliberation (*p* = 0.003 (Table 3)). The detailed results can be seen in Tables 3–5. Due to the lack of censored data, the Cox regression results were similar to those of the Table 5 model and are therefore not reported.

**Table 3:** A linear mixed model considering all maxillary canines, with eruption age as the outcome variable.

Model	Coefficient	Cl 95%	*P*-value
Intercept	9.67	8.28–11.07	0.000
Bone graft	0.19	–0.14–0.53	0.248
Sex	0.61	–0.40–1.63	0.226
Deliberation	1.05	0.40–1.70	0.003
Angulation	0.06	0.04–0.08	< 0.001

**Table 4:** A linear mixed model considering maxillary canines in unilateral cleft patients, with eruption age as the outcome variable.

Model	Coefficient	Cl 95%	*P*-value
Intercept	10.24	8.73–11.76	< 0.001
Bone graft	0.24	–0.14–0.61	0.197
Sex	0.33	–0.73–1.39	0.519
Deliberation	0.94	–0.01–1.89	0.053
Angulation	0.06	0.03–0.09	< 0.001

**Table 5:** A linear mixed model considering bone grafted teeth, with time between bone graft and eruption are set as an outcome variable.

Model	Coefficient	CI 95%	*P*-value
Intercept	5.97	1.82–10.12	0.007
Bone graft age	–0.55	–0.99 –(–0.11)	0.017
Sex	0.31	–0.65–1.27	0.510
Deliberation	1.21	0.22–2.20	0.025
Angulation	0.06	0.02–0.09	0.010

## Discussion

According to Lehtonen et al., cleft patients are at risk of having the following dental problems: multiple missing teeth, hypodontia, tooth agenesis, ectopic, impacted and supernumerary teeth, microdontia, transposition of the maxillary canine and the premolar teeth, delayed dental development, as well as crown and root malformation [28].

A study by Ristaniemi et al., conducted among Finnish patients with maxillary canine impactions and their need for treatment concluded that canines with larger inclination are more likely to need treatment (*p* = 0.001) [26]. In the respective study, the mean maxillary canine angle among 179 treated canines was determined to be 14.4 degrees. Additionally, the more severe the impaction, the longer the tooth takes to erupt [29]. These findings are in line with the results from the present study. A longer eruption time leads to prolonged treatment time because the misaligned tooth is likely to need extensive orthodontic treatment to achieve correct positioning.

In our study, there was no statistical difference in canine eruption on the grafted alveolar cleft side compared to the non-grafted side. In 2008, Russel and McLeod concluded that based on canine position only, the non-cleft side and the cleft side before and after bone grafting both had the same risk for impaction [27]. However, considering all factors such as timing of the ABG and the presence of the lateral incisor, patients with alveolar clefts had a 20-time higher risk for impaction. Also, in our study, the canine erupted later on the bone-grafted cleft site despite not being statistically significant. As tooth eruption is based on regulated bone resorption and remodelling [30, 31], the lack of need for extensive resorption may lower the resistance for the erupting tooth. This finding may support other findings in this study as postponing the bone graft delays the canine eruption.

The use of allograft requires further research before it can be recommended. Our series used only autograft material as it is the gold standard. While studies using allograft material have been reported [32, 33], our study did not comprise allograft.

Surgical exposure or deliberation of the maxillary canine is indicated when the maxillary canine fails to erupt or does not progress. While the eruption of the canine is not progressing, the tooth is embedded completely or incompletely in bone and/or mucosa. Therefore, it is logical for the canine to progress its eruption after deliberation. Addressing factors such as the maxillary canine position and angulation as well as providing supportive treatment such as extraction of an overlying obstructing deciduous tooth or surgical deliberation early enough may ease or facilitate the eruption of the respective impacted tooth

According to Kaura et al., there is a lack of evidence to support bone grafting at a specific age [34]. The grafting of the alveolar cleft site between 4 and 7 years of age has a significantly lower rate, requiring regrafting as compared to late-timed bone grafts. In patients who have had an alveolar bone graft between 11 and 13 years of age as many as 50% needed a secondary graft later on [35]. In the OCC, an ABG was in the majority of cases performed between 9 and 12 years of age, and in our study population, no additional bone grafts have been needed. The follow-up time window in terms of re-grafting procedure was until the maxillary canine was erupted. However, the Oulu protocol follows up patients until 18 years of age. If orthognathic surgery is indicated, the follow-up time is longer to provide retention after surgery. Mundra et al. recently reviewed 15 publications related to the timing of the ABG. Early mixed dentition bone grafting showed better long-term outcomes in 66.7% of the studied articles [36].

In a review article by Carbullido et al., a total of 24 research articles published between 1970 and 2019 reported that early ABG resulted in poor long-term survival. According to this review, an early ABG resulted in poor long-term survival. The findings were associated with maxillary underdevelopment, maxillary retrusion, poor occlusal outcomes and increase in crossbites, therefore not being recommended for clinical practice [37]. According to our study, early ABG seems to have no benefit in facilitating the canine eruption. The results may be limited due to the differences between methods, the surgeon’s skills, the outcome measurements and interpretation.

## Conclusion

In our study, the permanent maxillary canine eruption was reported to be slightly delayed after the ABG on the grafted site compared to the non-grafted site in cleft patients. The maxillary canine typically emerged about a year after it has been surgically exposed and deliberated. The maxillary canine angulation had an important role in the eruption. The early bone graft had no early benefit in terms of the timing of canine eruption. It is important to acknowledge the position and angulation of the erupting canine to plan orthodontic and possible surgical treatment that is well timed.
